# Aeration and agitation in hydroponic culture have detrimental effects on iron uptake

**DOI:** 10.3389/fpls.2025.1675950

**Published:** 2025-11-25

**Authors:** Noah James Langenfeld, Bruce Bugbee

**Affiliations:** Crop Physiology Laboratory, Department of Plants Soils & Climate, Utah State University, Logan, UT, United States

**Keywords:** corn, dissolved oxygen, iron chlorosis, sunflower, tomato

## Abstract

Aeration in deep-flow liquid hydroponics provides oxygen for respiration, but even gentle movement from solution agitation can alter the beneficial rhizosphere. Here we report the detrimental effects of bubbling-induced agitation of the rhizosphere on iron uptake and chlorosis of tomato, sunflower, and corn. We grew each species in deep-flow liquid hydroponics with aeration rates from 0 to 2 liters per minute and in a peat-based soilless media, which allowed plants to develop an undisturbed rhizosphere. All three species had ample iron and chlorophyll in soilless media with the same nutrient solution and pH as in liquid hydroponics. Conversely, chlorophyll and iron uptake were dramatically reduced in hydroponic sunflower and corn by gentle agitation of the solution. Tomato, however, was minimally affected by solution agitation. These results indicate that minimizing solution agitation allows the formation of a beneficial rhizosphere. Collectively, these studies demonstrate that controlled agitation might be used to alter root boundary layer thickness and thus quantify rhizosphere effects on nutrient uptake and growth.

## 1 Introduction

The chemical and microbial benefits of the rhizosphere are well studied in soils, but the rhizosphere is fragile in liquid culture and can be disrupted by bubbling for aeration.

Rhizosphere formation is critical to iron solubility and acquisition. Iron is essential to chlorophyll synthesis, but it is rapidly oxidized from ferrous iron (Fe^2+^) to ferric iron (Fe^3+^), which precipitates as insoluble ferric hydroxide (Kobayashi and Nishizawa, 2012). Interveinal chlorosis is a common symptom of reduced chlorophyll production from iron deficiency, which leads to decreased growth (Korcak, 1987). Plants excrete protons (strategy I) or phytosiderophores (strategy II) into the rhizosphere to help reduce ferric iron or chelate it to increase uptake (Römheld and Marschner, 1986; Marschner and Römheld, 1994). Dicotyledonous plants (sunflower and tomato) typically use strategy I, while monocots (corn) typically use strategy II.

The bulk solution pH and the rhizosphere pH have well-characterized effects on iron oxidation and availability (Marschner and Marschner, 2012). Jacobson and Oertli (1956) first reported slight chlorosis in aerated, hydroponic sunflower plants at a bulk pH of 5 and severe chlorosis in plants grown at pH 7. Synthetic chelates are now a standard component of hydroponic nutrient solutions and interact with pH to improve iron bioavailability and acquisition (Barton and Abadia, 2006). Mengel and Geurtzen (1988) grew hydroponic corn with iron chelated with ethylenediaminetetraacetic acid (Fe-EDTA) and observed severe chlorosis when using 100% nitrate-nitrogen and no chlorosis when using 100% ammonium-nitrogen, which they linked to changes in solution pH caused by the form of nitrogen uptake. Kosegarten et al. (1998) measured significantly reduced chlorophyll concentrations in hydroponic sunflower when using Fe-EDTA at pH 6.5 compared to pH 4, even though EDTA typically holds iron up to pH 7.

High phosphorus concentrations in the root-zone can form insoluble iron phosphates, which further decrease iron availability. Elliott and Läuchli (1985) reported iron chlorosis in hydroponic corn when grown with a phosphorus concentration of 100 µM and 25 µM, but not when grown with 5 µM phosphorus. Parry and Bugbee (2017) showed that reducing the phosphorus concentration from 700 µM to 70 µM increased chlorophyll concentration in hydroponic corn and improved growth and iron uptake.

Iron uptake efficiency varies widely among species. Brown and Jones (1976) studied hydroponic plants and found that tomato was more iron-efficient than soybean, which was more iron-efficient than corn. Sorghum was the most iron-inefficient. Cultivars within species also had varying degrees of iron efficiency. Zaharieva (1986) later analyzed the iron deficiency of plants grown in soil and found peanut to be more iron-efficient than soybean, which was more iron-efficient than corn. Campbell and Nishio (2000) studied sugar beet growth in hydroponics and found a nearly 2-fold decrease in chlorophyll concentration after 6 days for an iron-inefficient cultivar compared to an iron-efficient cultivar. Among strategy II plants, barley, wheat, and corn are generally more iron-efficient than rice and sorghum because they have higher rates of phytosiderophore release (Zuo and Zhang, 2011).

Maintaining a low pH, low phosphorus concentration, and using appropriate chelates can compensate for the reduced rhizosphere that occurs in liquid hydroponics.

### 1.1 Tolerance to low oxygen in hydroponics

The dissolved oxygen (DO) at saturation at 25°C, sea-level pressure, and low salinity is 8 mg per L, but it decreases to 6 mg per L at higher elevations (lower pressures) and warmer temperatures (Langenfeld and Bugbee, 2021). Without bubbling or solution flow, DO quickly decreases to less than 1 mg per L in planted systems. The absolute oxygen concentration in solution determines bioavailability, but some studies report only the percent of saturation without the solution temperature and atmospheric pressure.

Species vary in their tolerance to DO concentrations below saturation. Zeroni et al. (1983) found a decrease in tomato growth from 65 to 20% DO saturation, but they had no saturated DO control. Everard and Drew (1989) found that some hydroponic sunflower plants wilted when aeration was removed while others suffered no detrimental effects. Chun and Takakura (1994) reported the highest root growth in lettuce when nutrient solutions were saturated with oxygen at 8.3 mg per L, and reduced growth in over- or under- saturated conditions. Conversely, Goto et al. (1996) found no difference in lettuce growth between DO concentrations from 25 to 200% saturation (2 to 16 mg per L). Roosta (2024) found no difference in bell pepper growth as DO increased from 1.8 to 5.3 mg per L, but observed increased chlorophyll concentrations and photosynthetic rates at higher DO concentrations.

### 1.2 Aeration in liquid hydroponics

Effects of aeration intensity in liquid hydroponics are less well studied when compared to DO concentrations. Baiyin et al. (2021) reported no difference in Swiss chard growth as aeration intensity increased from 0.5 to 4 liters per minute (LPM) and the DO concentration remained above 6 mg per L. The lowest aeration rate in their study (0.25 LPM) caused the DO concentration to fall below 6 mg per L and led to decreased growth. Growth was also reduced at their highest aeration rate (8 LPM) despite the DO remaining above 6 mg per L, which they attributed to excessive root-zone agitation.

While rice is one of the most flood-tolerant (low oxygen) crop species, aeration can increase yield. Xu et al. (2013) found an increase in the root length, number, and volume when rice was aerated at 5 L per minute compared to a control with no aeration. Naznin et al. (2010) found a 4.3-fold increase in the fresh weight of hydroponic garlic bulbs in an aerated nutrient solution compared to a non-aerated control. Kratky (2005) studied lettuce growth in deep-water culture hydroponics without aeration and reported larger yields when container lids were at a fixed height over the nutrient solution compared to when the lid was floated on the solution surface. He attributed this to oxygen diffusion into the solution from the space between the nutrient solution and the lid.

### 1.3 Objective

Our objective was to determine if reduced hydroponic aeration and agitation affects iron uptake, and chlorophyll concentration. We hypothesized that chlorosis severity would increase, and iron uptake would decrease with increasing root-zone agitation caused by aeration intensity.

## 2 Methods

### 2.1 Plant material

Sunflower (*Helianthus annus*) and corn (*Zea mays*) seeds were soaked in a solution of 0.5% sodium hypochlorite (bleach) for 1 min and then thoroughly rinsed with tap water. The seeds were placed on germination paper (blue blotter, Seedburo Equipment Company, Des Plaines, IL, USA) soaked in tap water and set in a closed acrylic germination box for 2 d. The temperature was maintained at 25°C for the duration of the germination. Tomato (*Solanum lycopersicum*) seeds were placed directly on pre-soaked germination paper in a germination box without pretreatment. After germination, seeds were transferred to a slant board for 5 d using the method described in Langenfeld and Bugbee (2022). Seedlings were selected for root growth uniformity and transplanted into neoprene cloning collars in an extruded polystyrene sheet suspended over separate 8 L hydroponic containers (2-gallon black buckets with a 20.6 cm solution depth) in a glass greenhouse.

### 2.2 Environmental conditions

The greenhouse temperature was maintained at 25°C during the day and 21°C during the night. Supplemental LED lighting on a 16 h photoperiod ensured the photosynthetic photon flux density remained above 400 µmol m^-2^ s^-1^ for a daily light integral (DLI) above 25 mol m^-2^ d^-1^. The relative humidity averaged 50% ± 20% throughout the studies. The daytime CO_2_ concentration averaged 700 µmol mol^-1^ in trials one and two and 450 µmol mol^-1^ in the following trials.

### 2.3 Nutrient solution

The hydroponic nutrient solution for sunflower and tomato contained 1.5 mM calcium nitrate tetrahydrate, 2 mM potassium nitrate, 0.4 mM monopotassium phosphate, 0.8 mM magnesium sulfate heptahydrate, 0.6 mM potassium silicate, 7 µM iron-DTPA (diethylenetriaminepentaacetic acid), 3 µM manganese-EDTA (ethylenediaminetetraacetic acid) disodium hydrate, 3 µM zinc chloride, 40 µM boric acid, 4 µM copper-EDTA disodium, 0.1 µM sodium molybdate dihydrate, and 0.1 µM nickel (II) chloride hexahydrate. Nitric acid was added to a concentration of about 1.5 mM to titrate the solution to an initial pH of about 5.4. This same nutrient solution was used throughout each individual trial – none was discarded until after harvest. Containers were replenished with the same nutrient solution as needed throughout plant growth to ensure 90% to 100% of the original volume was maintained using the principles described in Langenfeld et al. (2022).

The nutrient solution was modified to increase iron availability for the trial with corn ‘Early Sunglow’. The nutrient solution contained decreased monopotassium phosphate (0.05 mM) and replaced the iron-DTPA with 5 µM ferric chloride and 50 µM iron-HEDTA (hydroxyethylethylenediaminetriacetic acid) as described in Bugbee and Langenfeld (2022). The pH was decreased to 5.0 using additional nitric acid. All other nutrients and concentrations remained the same.

### 2.4 Aeration treatments

Each container was assigned to receive one of three aeration rates: no aeration, 0.5 liters per minute (LPM), or 2 LPM. These aeration rates corresponded to aeration intensities of 0, 0.063, and 0.25 L of air per L of nutrient solution per minute (LLPM). Each aeration rate was replicated three times in separate containers. Aeration was constant for twenty-four hours a day in each treatment and monitored through a rotameter using filtered ambient air (21% oxygen). The highest aeration rate was necessary to maintain the solution DO close to saturation. Air was distributed into the solutions from a 1-inch diameter linear polyvinyl chloride (PVC) manifold attached parallel to the bottom of each container. The manifold was 15 cm long and contained ten evenly spaced 2 mm holes for air to exit into the nutrient solution.

Seven trials were conducted in liquid hydroponics (**Table 1**). The cultivar and/or days to harvest varied in each trial. A buffer [(2-(N-morpholino)ethanesulfonic acid (MES), adjusted to pH 5.5 with potassium hydroxide] was added to each container for a final concentration of 5 mM in trials one, two, and three to stabilize pH as demonstrated by Bugbee and Salisbury (1985). During trials one, two, and five each container was spiked with 56 µmol of iron-HEDTA two days prior to harvest to test if the chlorosis was iron-induced.

**Table 1:** A summary of the growth time, iron-HEDTA chelate addition, and MES buffer addition in seven liquid hydroponic trials with sunflower, corn, or tomato.

Trial	Species	Cultivar	Days	Iron-HEDTA pre-harvest	MES added
1	Sunflower	Teddy Bear	25	Yes	Yes
2	Sunflower	Lemon Pixie	26	Yes	Yes
3	Sunflower	Teddy Bear	19	No	Yes
4	Sunflower	Mammoth	21	No	No
5	Corn	Butterfruit	15	Yes	No
6	Corn	Early Sunglow	17	No	No
7	Tomato	Celebrity	28	No	No

The ‘days’ column is the time under treatment after transplanting.

### 2.5 Plants grown in soilless media

Plants grown in soils and soilless media can develop rhizospheres because there is no mechanical disturbance from solution agitation. Three plants each of sunflower (cultivars ‘Teddy Bear’ and ‘Mammoth’), corn (cultivar ‘Bonus’), and tomato (cultivar ‘Celebrity’) were additionally grown in a greenhouse in square, 6 inch-diameter, 2.75 L containers filled with a soilless media mixture to examine growth stability without mechanical agitation from aeration. This mixture contained 90% peat moss and 10% rice hulls by volume and was amended with 1.63 g L^-1^ calcium hydroxide (to increase pH to about 5.5), 1 g L^-1^ wollastonite (Vansil W-10, to provide ample silica and increase pH to about 6.0), and 0.75 g L^-1^ wetting agent (AquaGro G, to increase wettability). Seeds were planted directly in the media at a 2 cm depth without pretreatment. The plants were well-watered throughout their growth using the original nutrient solution described in section 2.3. The rhizosphere was not mechanically disturbed in the soilless media. The ‘Teddy Bear’ sunflower plants were grown for 26 days, the ‘Mammoth’ sunflower plants were grown for 28 days, the ‘Bonus’ corn plants were grown for 24 days, and the ‘Celebrity’ tomato plants were grown for 29 days.

### 2.6 Data collection

#### 2.6.1 Chlorophyll concentrations

Leaf chlorophyll was measured at harvest using a chlorophyll content meter (model MC-100, Apogee Instruments, Logan, UT) in trials three through seven and in the plants grown in soilless media. Three points were measured and averaged on each of the two uppermost fully expanded leaves on each plant (n = 18 total points per aeration treatment). Only one leaf was measured during the corn ‘Early Sunglow’ trial (n = 9 points per aeration treatment).

#### 2.6.2 Anthocyanin content

The anthocyanin content (in ACI) was measured using an anthocyanin content meter (model ACM-200 plus, Opti-Sciences, Hudson, NH) before harvest of the hydroponic tomato trial using the same technique as the chlorophyll measurements.

#### 2.6.3 Dissolved oxygen concentrations

The DO concentration in the nutrient solution of each plant in trials four, six, and seven was measured immediately prior to harvest using an optical DO meter (model ProSolo ODO/T, YSI, Yellow Springs, OH). We used an optical DO meter to improve accuracy at low oxygen concentrations. This meter does not consume oxygen and thus does not need to be moved in the solution to achieve an accurate measurement. Galvanic and polarographic sensors consume oxygen, which requires moving the electrode and potentially introducing oxygen into solution. The solution temperature was 25.5°C, the air pressure was about 86 kPa, and the electrical conductivity was about 1 mS cm^-1^, which equated to a saturation concentration of about 6.9 mg per L (calculated using the DOTABLES program from the U.S. Geological Survey, https://water.usgs.gov/water-resources/software/DOTABLES/, accessed 1 July 2025).

#### 2.6.4 Plant harvest

Plants in liquid hydroponics were harvested 17 to 28 d after transplanting. During harvest, plants were removed from the containers, and the aboveground biomass (shoot) was separated from the roots. The fresh mass of each component was measured before drying at 80°C. A constant mass was reached after 3 to 5 d of drying and the dry mass was then measured.

#### 2.6.5 Tissue analysis

Leaf and root tissue nutrient concentrations were analyzed at harvest in trial three. Dry tissue was ground and digested in nitric acid and hydrogen peroxide [method B-4.25 in Gavlak et al. (2005)]. Solutions were then analyzed using inductively coupled plasma optical emission spectroscopy (ICP-OES) by the Utah State University Analytical Laboratory in Logan, UT.

#### 2.6.6 Statistical analysis

Dry mass in the ‘Teddy Bear’ sunflower treatments was analyzed using a linear regression model (lm) from the ‘stats’ package in R (version 4.0.5; Foundation for Statistical Computing, Vienna, Austria). The dry mass response in the other cultivars and the leaf tissue iron concentrations were not linear and were compared using ANOVA in R with a Tukey HSD *post-hoc* test.

## 3 Results

We observed severe chlorosis in sunflowers grown in gently aerated liquid hydroponics, but not in plants grown in soilless media with the same nutrients, pH, and iron-chelates.

### 3.1 Nutrient solution pH

The pH slightly increased over time in all trials. The MES buffer addition stabilized most solutions below pH 6 in trials one, two, and three. When the MES buffer was removed in trial four (sunflower ‘Mammoth’), the pH increased at a faster rate and increased with an increasing aeration rate. Plants with no aeration had a nutrient solution pH of about 6.5 at harvest, while those with aeration had a pH of 7.3 (0.5 LPM) and 7.5 (2 LPM) at harvest. In trial five (‘Butterfruit’ corn), the pH at harvest averaged 6.26 with no aeration, 6.56 with 0.5 LPM aeration, and 6.63 with 2 LPM aeration. In trial six (‘Early Sunglow’ corn), the pH at harvest averaged 6.88 with no aeration, 7.19 with 0.5 LPM aeration, and 7.51 with 2 LPM aeration. In trial seven (‘Celebrity’ tomato), the pH at harvest averaged 6.39 with no aeration, 6.97 with 0.5 LPM aeration, and 7.68 with 2 LPM aeration.

### 3.2 Chlorosis

**Figure 1:**
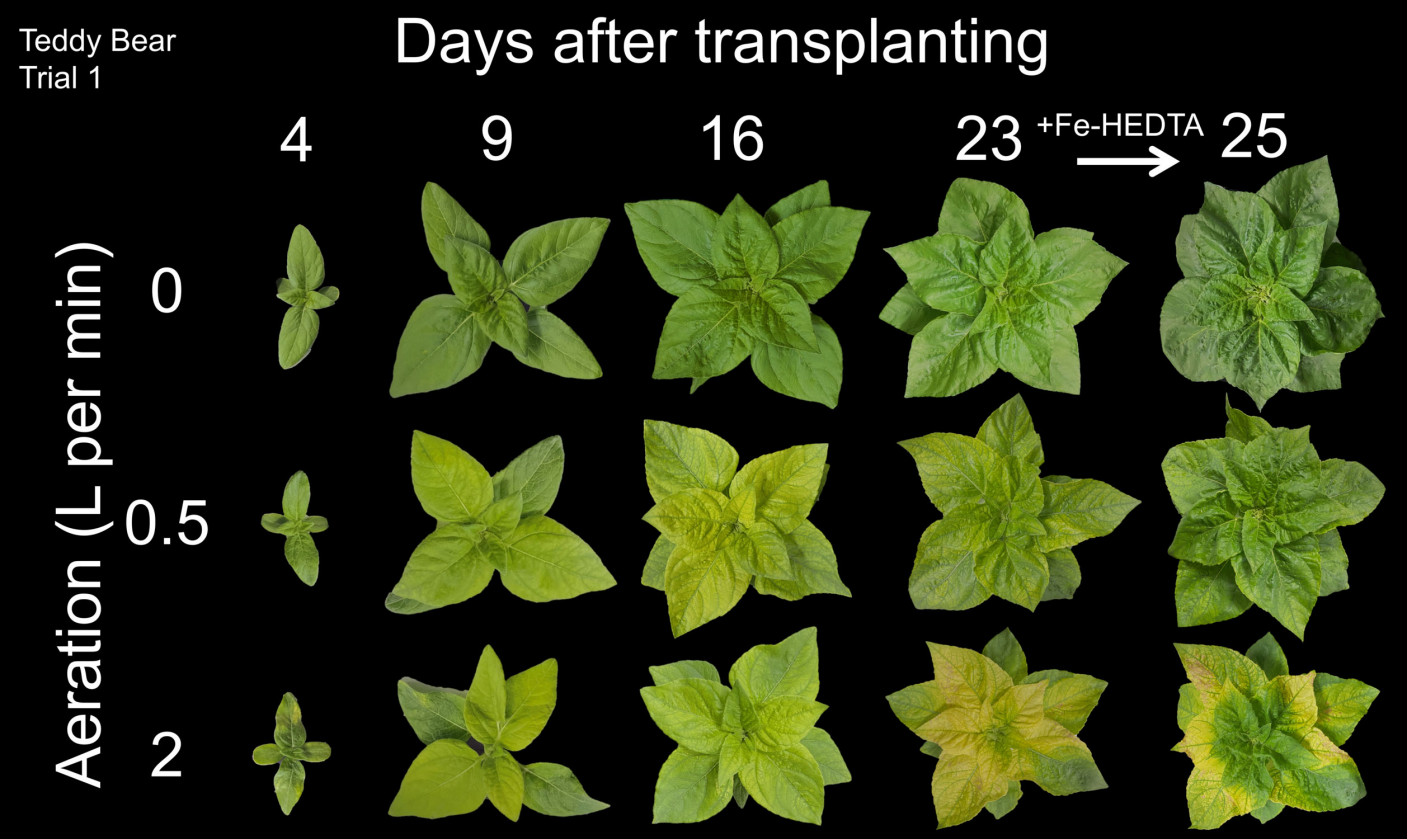
A top-down view of ‘Teddy Bear’ sunflower plants grown trial 1 in 8 L liquid hydroponic containers under aeration rates of 0, 0.5, and 2 liters per minute at five intervals after transplanting. Hydroxyethylethylenediaminetriacetic acid (HEDTA) iron chelate was added to all solutions 2 d before harvest. The same representative plant from each treatment was photographed throughout the trial.

**Figure 2:**
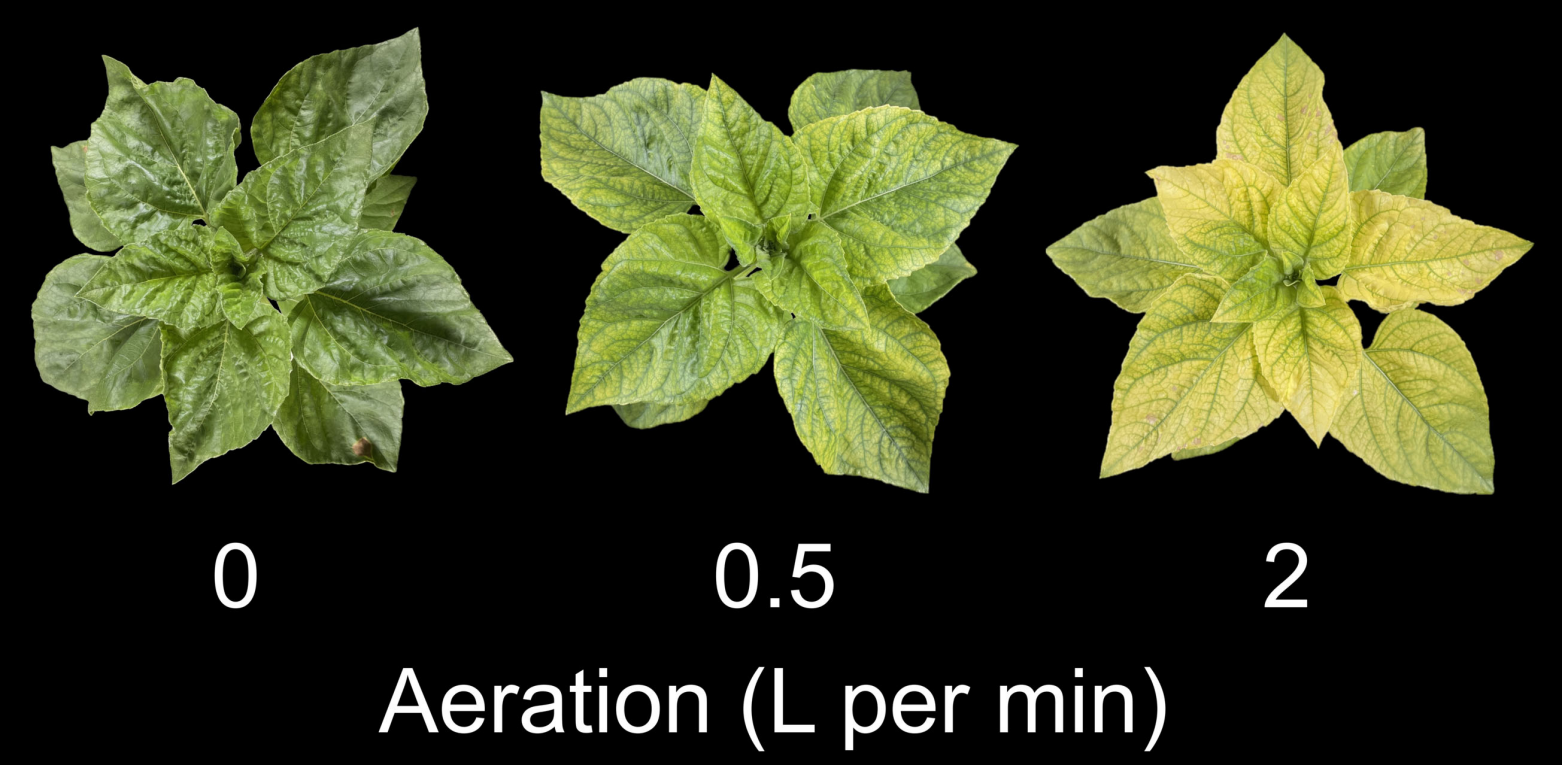
‘Teddy Bear’ sunflower plants grown in trial 3 in liquid hydroponics with aeration rates of 0, 0.5, and 2 liters per minute. Pictures were captured from representative plants at harvest.

All plants were green with no visible chlorosis following transplanting, but chlorosis rapidly appeared in sunflower plants grown with aeration rates of 0.5 or 2 LPM. Plants grown with aeration rates of 0.5 or 2 LPM turned green after the addition of iron-HEDTA chelate before harvest (**Figure 1**). Plants grown with no aeration remained green throughout the study and showed no signs of chlorosis in ‘Teddy Bear’ or ‘Mammoth’ sunflower. ‘Lemon Pixie’ sunflower plants grown with no aeration developed chlorosis, but at a slower rate than those grown at higher aeration rates. When plants were grown without the addition of iron-HEDTA chelate, plants with aeration were severely chlorotic at harvest (**Figure 2**).

Corn plants were light green in trial five (‘Butterfruit’ corn) regardless of aeration treatment but appeared to become more chlorotic under higher aeration rates. The nutrient solution was adjusted in trial six (‘Early Sunglow’ corn), which caused the plants to appear greener under no aeration than in trial 5. Chlorosis severity still increased with increasing aeration rate, which was similar to what was observed in sunflower.

None of the tomato plants were visually chlorotic. Plants grown under 2 LPM of aeration were smaller, had thicker leaves, and were visually darker and more purple-colored than those grown under no aeration or 2 LPM aeration.

### 3.3 Chlorophyll concentration and anthocyanin content

**Figure 3:**
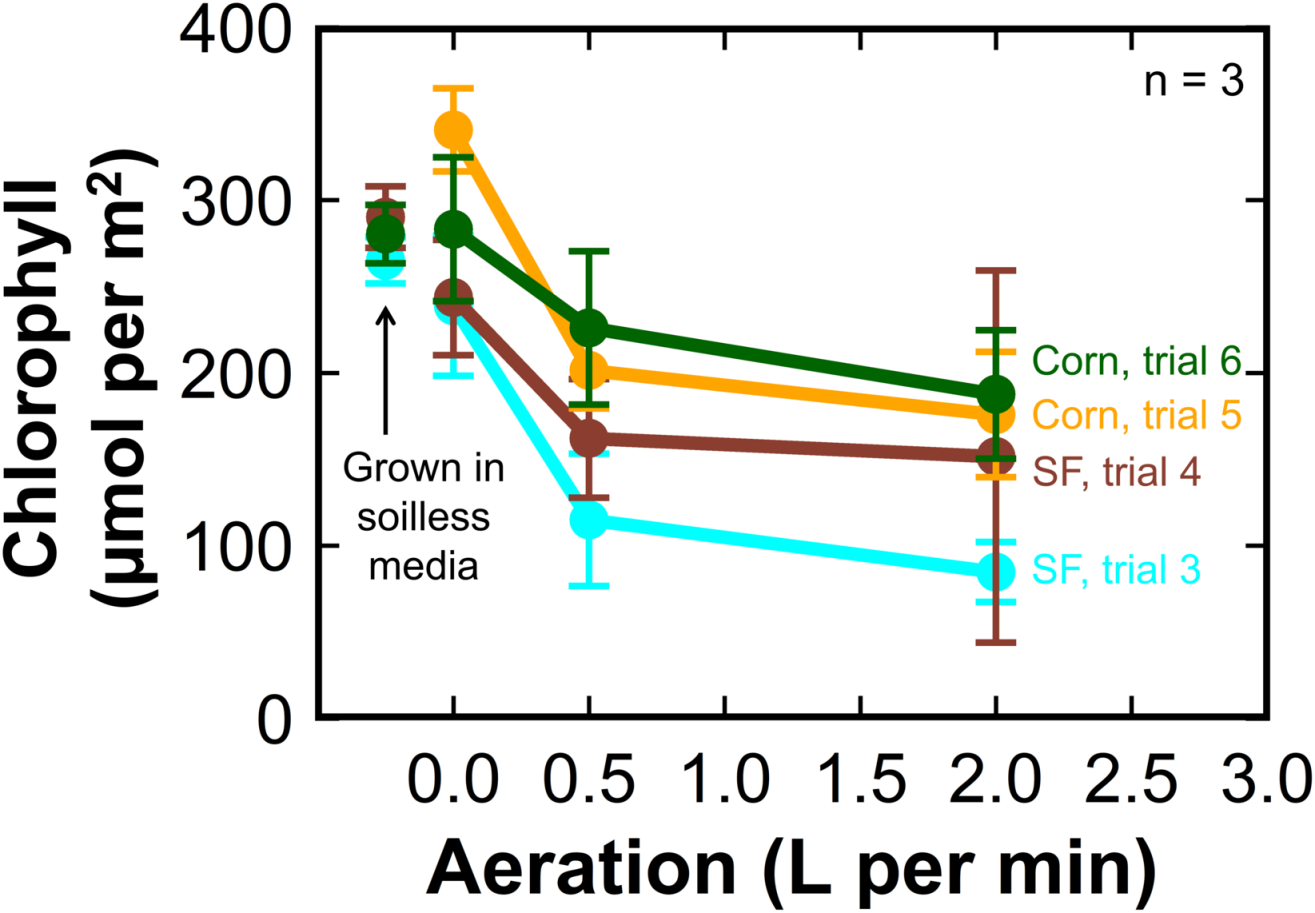
The effect of aeration rate on leaf chlorophyll concentration at harvest of hydroponic sunflower (cyan, trial 3, and brown, trial 4) and corn (yellow, trial 5, and green, trial 6). The chlorophyll concentrations in the plants grown in soilless media were similar to the corn and sunflower treatments without aeration. Error bars represent standard deviation, n = 3.

Sunflower and corn plants grown without aeration or agitation had chlorophyll concentrations similar to plants grown in soilless media (**Figure 3**). There was no difference in chlorophyll concentration in leaves between plants grown with 0.5 LPM or 2 LPM of aeration. Tomato plants grown in soilless media had equivalent chlorophyll concentrations to plants grown with 0.5 LPM and 2 LPM aeration in liquid hydroponics.

The anthocyanin concentration index (ACI) in tomato leaves at harvest was more than twice as high in the plants grown with no aeration (23.8 ± 2.8 ACI), compared to plants grown with 0.5 LPM (11.8 ± 0.9 ACI) and 2 LPM (10.8 ± 1.1) aeration.

### 3.4 Iron concentrations in leaf and root tissues

**Figure 4:**
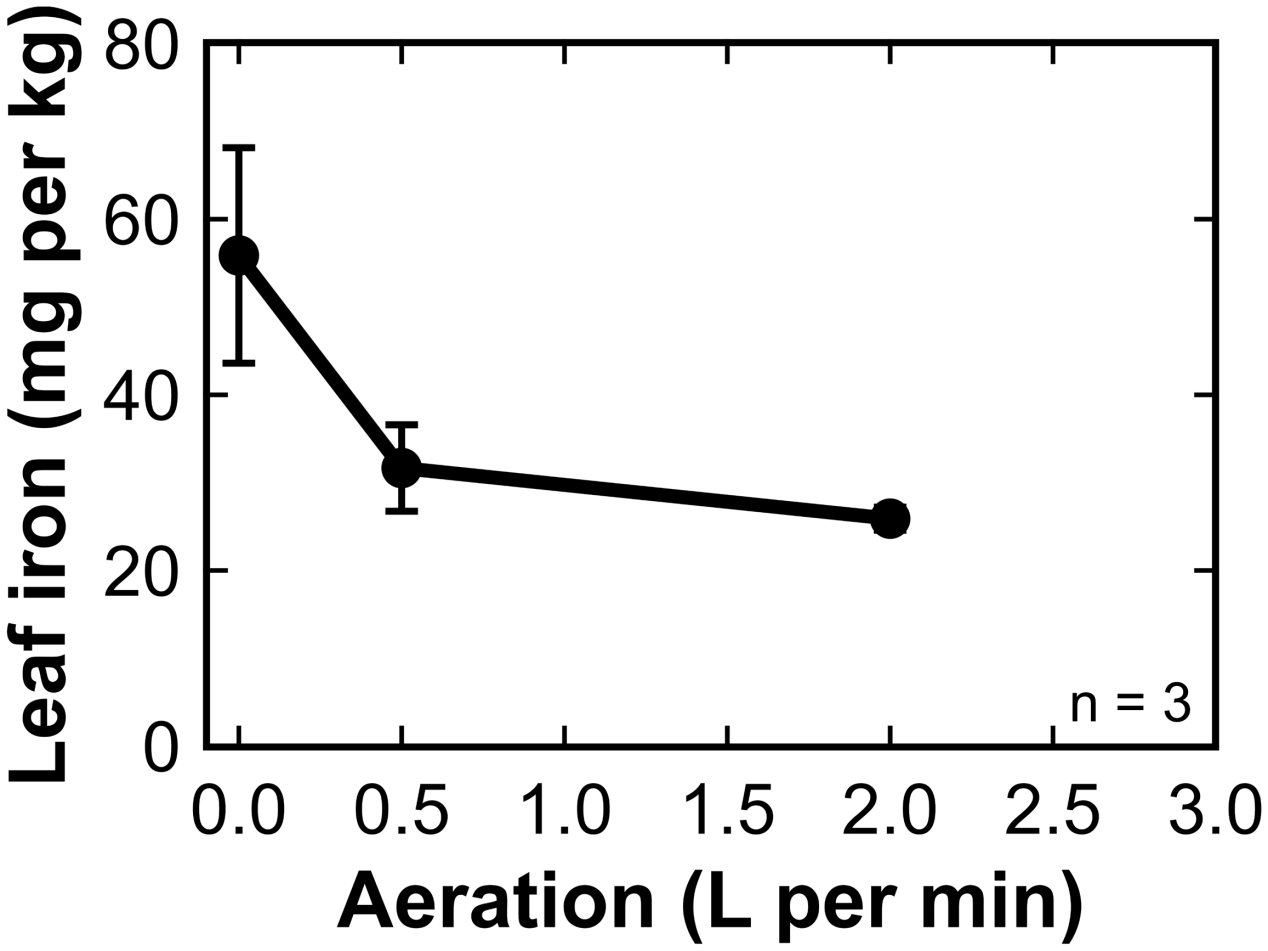
The concentration of iron in the dry leaf tissue of sunflower (‘Teddy Bear’ trial 3) grown in 8 L liquid hydroponic containers under aeration rates of 0, 0.5, and 2 liters per minute (LPM). Error bars represent standard deviation, n = 3.

The leaf tissue iron concentration of ‘Teddy Bear’ sunflower in trial three was significantly higher in plants grown under no aeration compared to plants grown with aeration (**Figure 4**).

Root samples from the three plants in each treatment were combined and a single sample was analyzed. Root iron followed the opposite trend to leaf iron. The root iron increased from 216 mg kg^-1^ at 0 LPM, to 260 mg kg^-1^ at 0.5 and 617 mg kg^-1^ at 2 LPM. Iron analysis typically indicates iron on roots and not iron in roots (Mengel and Geurtzen, 1988; Longnecker and Welch, 1990). This indicates that less iron was precipitated on root surfaces with less agitation in the solution and more of the rhizosphere iron was absorbed.

### 3.5 Dry mass

**Figure 5:**
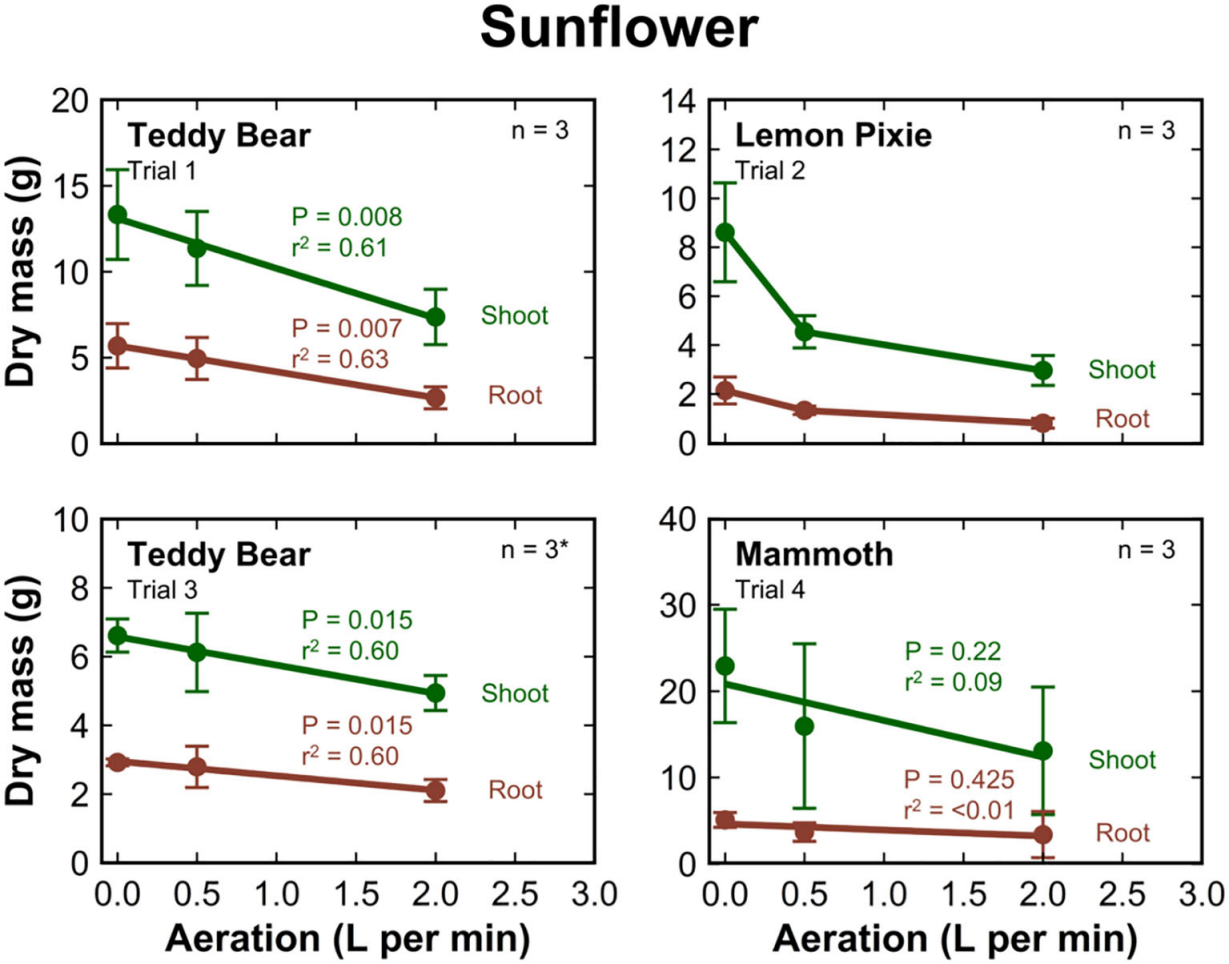
The dry shoot (green) and root (brown) mass of sunflower cultivars grown in 8 L liquid hydroponic containers under aeration rates of 0, 0.5, and 2 liters per minute. Error bars represent standard deviation, n = 3, except for the 0.5 LPM treatment in ‘Teddy Bear’ sunflower trial 3 where an outlier plant was removed from measurement.

The shoot and root mass decreased with increasing aeration rate in all sunflower cultivars (**Figure 5**). There was a significant decrease in the dry mass of ‘Teddy Bear’ sunflower in trial one (shoot, P = 0.008, r^2^ = 0.61; root, P = 0.007, r^2^ = 0.60) and trial three (shoot, P = 0.015, r^2^ = 0.60; root, P = 0.015, r^2^ = 0.60). The shoot and root mass of ‘Mammoth’ sunflower decreased with increasing aeration rate in trial four, but the decreases were not significant. There were also decreases in the shoot and root mass of ‘Lemon Pixie’ sunflower with increasing aeration rate, but these decreases were not linear and were not statistically analyzed.

**Figure 6:**
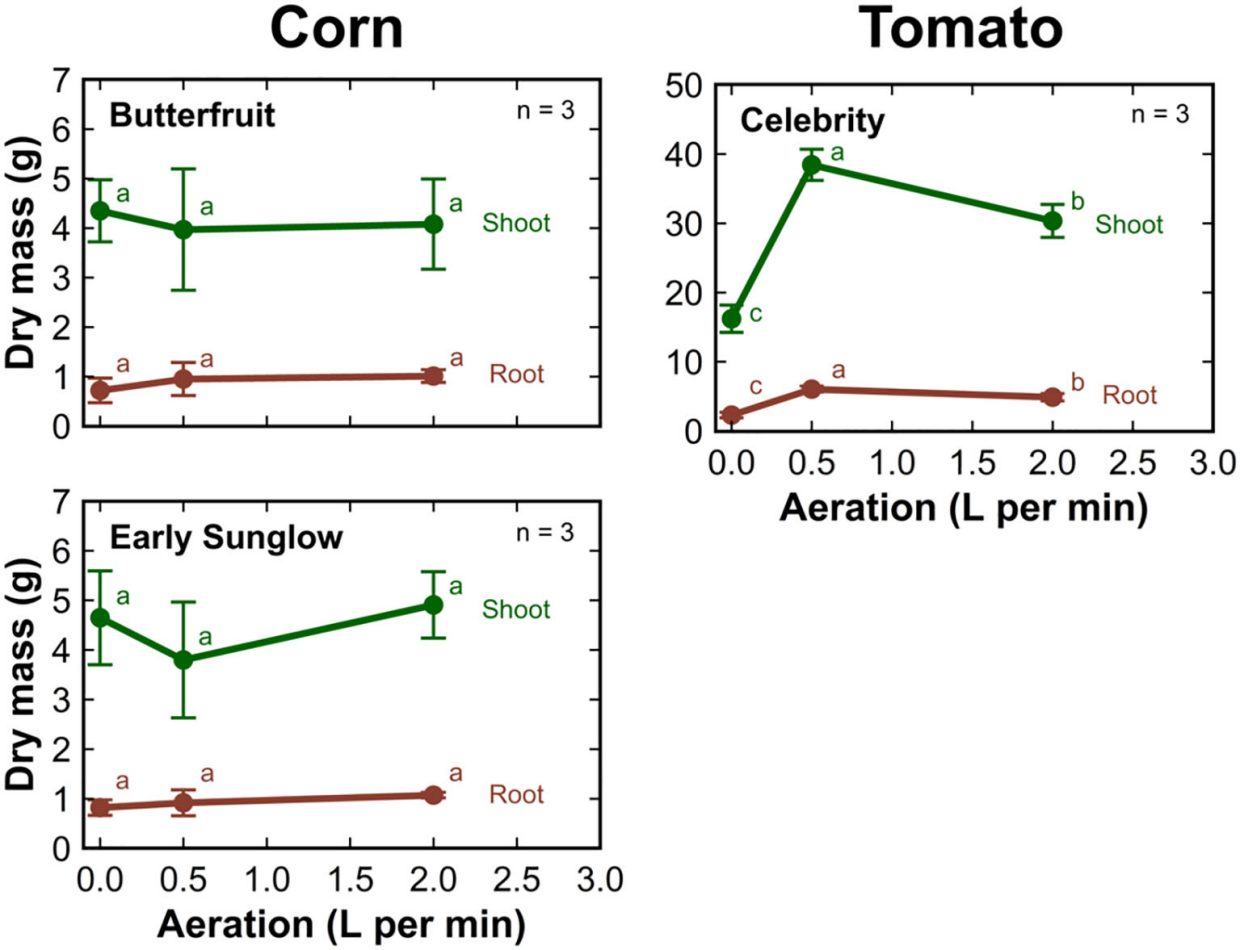
The dry shoot (green) and root (brown) mass of corn and tomato cultivars grown in 8 L liquid hydroponic containers under aeration rates of 0, 0.5, and 2 liters per minute. Error bars represent standard deviation, n = 3.

There was no significant change in the shoot or root mass of corn with increasing aeration rate (**Figure 6**). The dry shoot and root mass of tomato was the highest when grown at an aeration rate of 0.5 LPM. The shoot and root mass decreased by about 50% when plants were grown without aeration. Growth was reduced by about 25% when plants grew at an aeration rate of 2 LPM compared to an aeration rate of 0.5 LPM.

### 3.6 Dissolved oxygen concentrations

**Figure 7:**
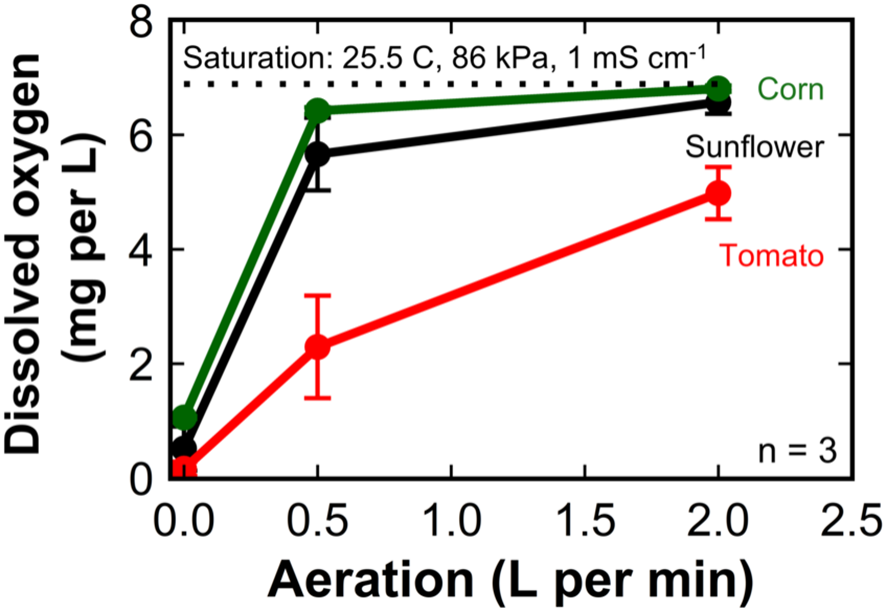
The dissolved oxygen concentration in the nutrient solution at harvest of sunflower, corn, and tomato grown in 8 L liquid hydroponic containers with aeration rates of 0, 0.5, and 2 liters per minute. Error bars represent standard deviation, n = 3.

We calculated the DO concentration at saturation to be 6.9 mg L^-1^ from the air pressure of 86 kPa, the solution temperature of 25.5°C, and the electrical conductivity of 1 mS cm^-1^. The DO concentration in the nutrient solution at harvest in the ‘Mammoth’ sunflower trial was 0.52 ± 0.39 mg L^-1^ (8% saturation) for plants grown under no aeration and increased to 6.57 ± 0.20 mg L^-1^ (95% saturation) for plants grown under an aeration rate of 2 LPM (**Figure 7**).

The DO concentration in the nutrient solution at harvest in the ‘Early Sunglow’ corn trial was 1.06 ± 0.03 mg L^-1^ (15% saturation) for plants grown under no aeration and increased to 6.81 ± 0.06 mg L^-1^ (99% saturation) for plants grown under an aeration rate of 2 LPM.

The DO concentration in the nutrient solution at harvest in the ‘Celebrity’ tomato trial averaged only 0.16 ± 0.13 mg L^-1^ (2% saturation) for plants grown under no aeration and increased to 4.98 ± 0.46 mg L^-1^ (72% saturation) for plants grown under an aeration rate of 2 LPM.

## 4 Discussion

### 4.1 Iron-induced chlorosis

There was a clear and consistent detrimental effect of increasing aeration and agitation on chlorosis in sunflowers. We confirmed that this was iron-induced chlorosis by observing greening one day after application of iron-HEDTA chelate. This chelate has a lower formation constant (21.0) for iron than DTPA (29.9) and thus more easily releases iron (Norvell, 2018). Preliminary results (unpublished) indicated that chlorosis was not chelate dependent as plants developed chlorosis regardless of chelate type.

It may be possible to supply chelated iron at a high concentration so that iron transformations in the rhizosphere are not necessary. Garousi et al. (2016a) grew sunflower and corn (2016b) in aerated hydroponics to study selenium toxicity, but reported no chlorosis when iron was supplied as 100 µM Fe-EDTA. This concentration is 14 times higher than the 7 µM concentration used in this study, which is commonly used in hydroponic nutrient solutions.

### 4.2 Chlorosis and pH

Nearly 50 years ago, Römheld and Marschner (1979) demonstrated the ability of sunflower to modify the rhizosphere by reducing pH. They concluded that sunflower was more efficient at iron acquisition than corn by growing plants with increasing concentrations of chelated iron. Mariotti et al. (1996) similarly found that corn was more sensitive to low iron concentrations than sunflower. However, we observed a higher sensitivity to rhizosphere disruption in sunflower than corn. Masalha et al. (2000) grew sunflower and corn in loam soil at a pH of 6.4 and observed a 4.1-fold decrease in chlorophyll concentration in sunflower under sterile conditions (no microbial activity) compared to a 1.3-fold decrease in corn, which they attributed to the ability of corn to produce phytosiderophores. Collectively, our results indicate that sunflower may be more iron-efficient in the field or in soilless media where it can acidify the rhizosphere without agitation.

Conversely, iron-induced chlorosis is increased by an alkaline pH (Marschner and Marschner, 2012). As expected, Jacobson and Oertli (1956) saw a decrease in total iron concentration in the leaf tissue of sunflowers grown in aerated hydroponic solutions with increasing pH. Similarly, Kosegarten et al. (1998) reported chlorotic hydroponic sunflowers grown at pH 6.5 with EDTA. We observed chlorosis and low chlorophyll contents even though the pH remained below 6 during the first three sunflower trials. This indicates that sunflowers are sensitive to iron-induced chlorosis in liquid hydroponics.

### 4.3 High iron concentrations in root tissues

There was a decrease in leaf tissue iron concentration, but an increase in root tissue iron with increasing chlorosis. These measurements are consistent with values reported in the literature. Mengel and Geurtzen (1988) found elevated iron in the root tissue of corn compared to leaf tissue. They measured iron in root tissue ranging from 400 to 1250 mg kg^-1^ compared to iron in young leaf tissue ranging from 84 to 164 mg kg^-1^. We suspected the increased root tissue iron to be precipitated iron in the root apoplast and not inside the root symplast. Longnecker and Welch (1990) measured increased iron in the root apoplast of soybean compared to the symplast, which they hypothesized served as a reserve to minimize future iron deficiency. Despite the high root iron concentrations seen in our study, sunflower was likely unable to mobilize the iron to the leaf tissue due to the high agitation mechanically disrupting the rhizosphere.

### 4.4 Oxygen stress and aerenchyma

An elevated anthocyanin content index (ACI) is often an indication of abiotic stress in plants (Chalker-Scott, 1999; Naing and Kim, 2021). We observed dark, purple-colored leaves in tomato plants grown with no aeration and measured an ACI more than double that of plants grown with aeration. The lack of DO in the root-zone may have led to oxygen stress in the tomato roots, which are known to be particularly sensitive to oxygen deprivation from flooding stress (Rao and Li, 2003). This could have also led to the darker colored leaves we observed in tomato grown with no aeration.

Corn was less susceptible to oxygen stress, which may have been due to its ability to form aerenchyma. Formation of these air-filled channels is common in many plants when they experience hypoxic conditions due to flooding (Yamauchi et al., 2013). Drew et al. (1979) were among the first to observe aerenchyma formation in corn and their formation is now well-known in cereal crops (Jackson et al., 1985; Evans, 2004). Mano et al. (2006) specifically found that corn plants under oxygen stress from flooded containers formed aerenchyma to aid in oxygen transport. Aerenchyma are not usually found in plants grown under non-flooded conditions but Konings and Verschuren (1980) found that corn developed aerenchyma even when grown in hydroponic nutrient solutions aerated at 0.3 LPM. These findings suggest that corn may form aerenchyma in many oxygen conditions and likely explain why we saw no difference in corn growth among aeration treatments.

While aerenchyma have been found in many monocots, their presence in dicots is less well studied. Kawase (1979) is one of the few to have demonstrated aerenchyma development in sunflower as a function of increased ethylene. Kawase and Whitmoyer (1980) additionally observed the formation of aerenchyma in both sunflower and tomato stems within two days of waterlogging the roots, but they did not test for aerenchyma in roots. Kawase (1981) then extended this research to show aerenchyma formation in sunflower, tomato, and bean under elevated ethylene. None of these studies, however, were conducted in deep flow hydroponics. The potential presence of aerenchyma in sunflower would explain why growth was not reduced under low DO concentrations. Aerenchyma formation in tomato may have been inadequate to deliver enough oxygen to maintain root respiration at the lowest aeration level. Despite reduced dissolved oxygen, the most rapid tomato growth occurred in the low aeration (0.5 LPM) treatment. This suggests that tomato might also benefit from reduced agitation in hydroponic culture.

Plants began turning green near harvest in the aerated treatments in the ‘Mammoth’ sunflower trial. The high growth rate of this cultivar near harvest led to a rapid depletion of the solution volume (1 to 2 L per day), which meant unoxidized iron was being added to the nutrient solutions more frequently. Raju and Marschner (1973) also reported a ‘regreening’ of hydroponic sunflowers following a period of chlorosis that they linked to a decrease in pH and an increase in iron solubility, but we never observed a decrease in bulk solution pH.

There may be a link between tolerance to low oxygen and iron acquisition ability: species with a high tolerance to low oxygen may be better at accessing iron.

### 4.5 Redox potential and iron oxidation

The high aeration rates and increased DO concentrations would increase redox potential and could oxidize iron to less bioavailable forms. However, Boyd (2000) demonstrated only a small effect of DO on redox potential (ORP). At pH 7, the ORP decreases less than 20 mV as the DO decreases from 8 to 0.5 mg per L. Any contribution of changing ORP as a function of DO on iron oxidation would have therefore been minor.

### 4.6 Future work

A limitation of this study is the lack of separation between mechanical agitation and oxygenation. We are currently conducting studies to provide oxygen in solutions with minimal disruption of the rhizosphere boundary layer. Using high-quality air stones to decrease bubble diameter maintains DO with minimal solution agitation.

Additional work might use low-agitation hydroponics to examine microbe-dependent iron acquisition mechanisms unique to sunflower. Microbial communities are reduced in liquid hydroponic systems but they can be established in soil and soilless media where iron acquisition is greatly increased.

## 5 Conclusion

Our results are the first to link chlorosis to agitation from aeration of crops grown in liquid hydroponic culture. We found iron-induced chlorosis in the leaves of sunflower and corn grown in pH controlled, aerated liquid hydroponics, but not in tomato. This chlorosis led to a decrease in growth of sunflowers with an increasing aeration rate, which was linked to the reduced iron uptake due to the continuous solution agitation. The lack of agitation in plants receiving no root-zone aeration led to the highest sunflower growth, and low agitation increased tomato growth. This suggests that reduced aeration rates may favor the formation of a rhizosphere and increase iron uptake in liquid hydroponics. These findings facilitate the use of precisely controlled agitation to alter the rhizosphere because oxygen concentration can be maintained in hydroponics with minimal agitation. Controlled agitation offers a powerful new technique to study the value of the rhizosphere on nutrient uptake and plant growth.

## Data Availability

The original contributions presented in the study are included in the article/supplementary material. Further inquiries can be directed to the corresponding author.
